# A Review on Current COVID-19 Vaccines and Evaluation of Particulate Vaccine Delivery Systems

**DOI:** 10.3390/vaccines9101086

**Published:** 2021-09-27

**Authors:** Sarthak M. Shah, Hashem O. Alsaab, Mutasem M. Rawas-Qalaji, Mohammad N. Uddin

**Affiliations:** 1 College of Pharmacy, Mercer University, Atlanta, GA 31207, USA; Sarthak.Modi.Shah@live.mercer.edu; 2 Department of Pharmaceutics and Pharmaceutical Technology, Taif University, P.O. Box 11099, Taif 21944, Saudi Arabia; h.alsaab@tu.edu.sa; 3 College of Pharmacy, University of Sharjah, Sharjah 26666, United Arab Emirates; mqalaji@sharjah.ac.ae; 4 Research Institute for Medical and Health Sciences, University of Sharjah, Sharjah 26666, United Arab Emirates; 5 Dr. Kiran C. Patel College of Allopathic Medicine, Nova Southeastern University, Fort Lauderdale, FL 27272, USA

**Keywords:** COVID-19, vaccines, SARS-CoV-2, pandemic, oral particulate vaccine

## Abstract

First detected in Wuhan, China, a highly contagious coronavirus, severe acute respiratory syndrome coronavirus-2 (SARS-CoV-2), also known as COVID-19, spread globally in December of 2019. As of 19 September 2021, approximately 4.5 million people have died globally, and 215 million active cases have been reported. To date, six vaccines have been developed and approved for human use. However, current production and supply capabilities are unable to meet global demands to immunize the entire world population. Only a few countries have been able to successfully vaccinate many of their residents. Therefore, an alternative vaccine that can be prepared in an easy and cost-effective manner is urgently needed. A vaccine that could be prepared in this manner, as well as can be preserved and transported at room temperature, would be of great benefit to public health. It is possible to develop such an alternative vaccine by using nano- or microparticle platforms. These platforms address most of the existing vaccine limitations as they are stable at room temperature, are inexpensive to produce and distribute, can be administered orally, and do not require cold chain storage for transportation or preservation. Particulate vaccines can be administered as either oral solutions or in sublingual or buccal film dosage forms. Besides improved patient compliance, the major advantage of oral, sublingual, and buccal routes of administration is that they can elicit mucosal immunity. Mucosal immunity, along with systemic immunity, can be a strong defense against SARS-CoV-2 as the virus enters the system through inhalation or saliva. This review discusses the possibility to produce a particulate COVID vaccine by using nano- or microparticles as platforms for oral administration or in sublingual or buccal film dosage forms in order to accelerate global vaccination.

## 1. Introduction

The first coronavirus was discovered by Tyrell and Bynoe in the 1960s [1]. In 1967, McIntosh et al. reported the finding of several other strains of the virus [2]. Due to their bulbous, crown-shaped surface projections, the name “coronavirus” was announced as a new genus to describe these viruses [1,3]. Decades later, coronaviruses made headlines again when the first instance of the 2019 novel coronavirus was detected in Wuhan, China [4]. Later that month, a travel-related coronavirus case was detected in Illinois, USA [5]. On 9 January 2020 a news outlet reported that laboratory tests on samples from patients found 15 positive results of the new-type coronavirus, and this new-type, SARS-CoV-2, was isolated from one patient’s samples. The laboratory results showed SARS-CoV-2 had caused viral pneumonia in 59 cases [6].

**Figure 1:**
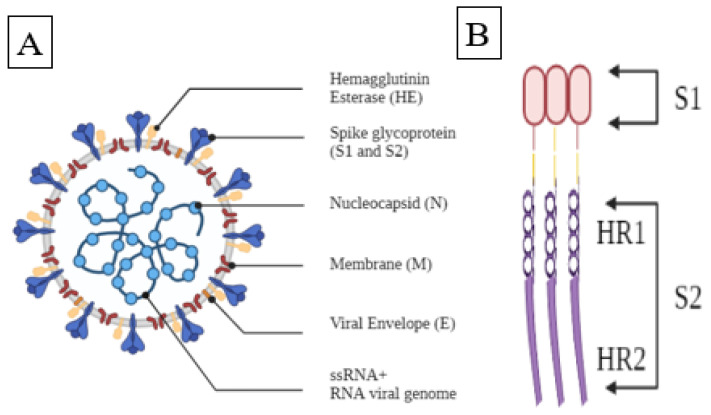
(**A**). Structure of SARS-CoV-2, (**B**). Spike protein of SARS-CoV-2.

After a brief period, the virus swiftly spread globally causing fear and chaos around the world. According to the Centers for Disease Control and Prevention (CDC), there are seven types of coronaviruses which can infect humans [7]. The coronavirus SARS-CoV-2 can primarily be transmitted via inhalation and secondarily transmitted via direct contact to a surface or object where the virus exists [8]. The virus then uses its spike proteins to enter a cell. Figure 1 shows the structure of SARS-CoV-2 and the spike protein.

**Figure 2:**
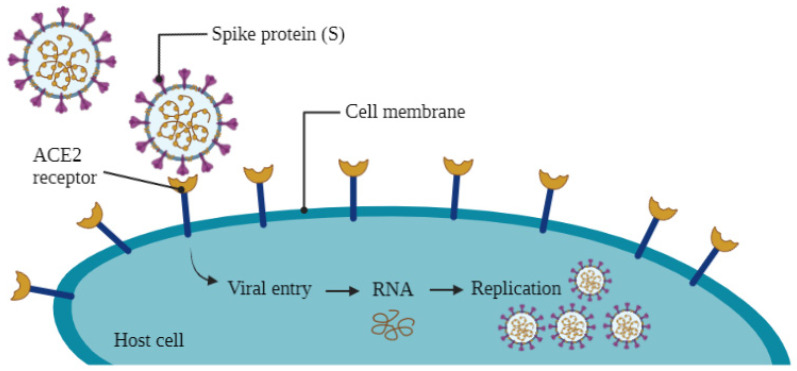
How the SARS-CoV-2 virus enters and infects human cells.

The spike protein of the virus attaches to the ACE2 receptor of the cell. This vital interaction (Figure 2) between the spike protein and ACE2 receptor allows the virus to adhere to and enter the cell [9]. Interestingly, another receptor, CD209L (L-SIGN), has also been discovered to be used by the SARS-CoV-2 virus to enter human cells [10]. The fusion of the virus to human cells is carried out by conformational changes to the receptor spike protein. Factors that trigger this conformational change include receptor binding, an acidic pH, or proteolytic activation [11]. Once inside the cell, the virus fuses with a vesicle and releases its stored RNA. Once released, the RNA is translated into proteins. Next, the virus is assembled in the cell’s cytoplasm and is eventually released from the cell [12]. Once the virus is released, special cells named Antigen-Presenting Cells (APCs) have the core responsibility of capturing the antigen or virus and presenting a piece of it on their surface [12]. These APCs are dendritic cells, macrophages, B cells, or Langerhans cells [13]. After a viral peptide is presented on the surface of an APC, a T-helper cell binds to this viral peptide, activating the T cell. This T cell can then continue to activate a B cell to produce anti-SARS-CoV-2 antibodies. In addition, the T-helper cell can mark the virus for destruction with help from cytotoxic T cells [12].

Developing a vaccine is an effective approach to combatting this lethal virus. Laboratories all around the world are developing vaccines through a variety of platforms. Such platforms include vaccines based on nucleic acid (DNA or RNA), proteins (protein subunit or virus-like particles), viral vectors (replicating or nonreplicating), and weakened or inactivated viruses [12]. Although some vaccines show favorable results, it is vital to cautiously evaluate every SARS-CoV-2 candidate to ensure that the vaccine does indeed show proper effectiveness and safety before being distributed to millions of people worldwide. Currently, two doses are needed for the Pfizer (BNT162b2), Moderna (mRNA-1273), and AstraZeneca (AZD1222) vaccines [14]. However, experts are currently speculating whether a third booster shot may be needed. Moderna reported that a third dose was effective against variants in South Africa and Brazil. This suggests a third booster may be needed to fight unwelcome variants in some countries. The need for an additional booster shot only increases the pressure on vaccine manufacturers, who are already struggling to provide enough vaccines. Moreover, there are some adverse effects that have been observed, most prominently blood clotting [15]. Additionally, the vaccine developed by AstraZeneca, ChAdOx1 nCov-19, causes the unusual, rare development of immune thrombotic thrombocytopenia [16]. Due to these limitations, mass application of these vaccines around the world is severely hindered. The ultimate goal of vaccination is to ensure the production of strong and lasting immune responses after a single dose of antigen without the need for booster doses [17]. In order to ensure the quality and magnitude of an immune response needed to prevent illness, it is highly important that the immune system is presented with antigens in a sufficient quantity at the right locations of encounter with targeted pathogens [18,19].

Particulate COVID vaccine is a vaccine that can be developed by using nano- or microparticles as platforms. Particles consist of an active principle (drug or biologically active material) that is dissolved, entrapped, or encapsulated [20]. Microparticles and nanoparticles can be utilized as drug carriers into which drugs or antigens may be incorporated in the form of solid solutions or solid dispersions. Nano- or microparticles have been shown to enhance the delivery of certain drugs across several natural and artificial membranes. Although the currently marketed mRNA vaccines are in particulate form, these are administered via the intramuscular route. This route of administration is at a disadvantage due to decreased patient compliance associated with injections. Therefore, particulate vaccines that can be administered by some other, painless route such as buccal or sublingual can be a better option for this vaccine. In this article, we will review some of the current COVID-19 vaccine platforms and discuss their limitations. We will further explore the feasibility of developing a COVID vaccine that can be administered orally, sublingually or via the buccal route by utilizing nano- or microparticles as a platform.

## 2. Current COVID-19 Vaccine Platforms

Many pharmaceutical industries and research organizations (both governmental and non-governmental) are racing to manufacture vaccines to fight the COVID-19 pandemic. These research institutions use several platforms for vaccines. Some of the vaccines have already been introduced for public vaccination and some are still in the pipeline. Among these platforms, mRNA-based, recombinant protein-based, viral vector-based, bacterial vector-based, and plasmid DNA-based vaccines are the most notable. Some of these vaccine platforms are described below.

### 2.1. mRNA Based COVID Vaccines

The major and leading pharmaceutical industries in the USA, such as Pfizer and Moderna, are using mRNA-based vaccines to prevent COVID-19. AstraZeneca’s vaccine is different from the other COVID vaccines in that the formulation uses double stranded DNA, rather than mRNA [21]. The main advantage of this platform is quick preparation, which is very crucial during a pandemic situation. Normally a vaccine takes about 10 to 15 years from the beginning of its preparation to come to the market. However, using mRNA technology these industries have accelerated the process and have marketed much-needed vaccines. The difference in production speed of these vaccines is owing to the fact that viral vaccine manufacturing relies on animal cell biology, while RNA manufacturing is a cell-free biochemical process performed with synthetic enzymes [22]. Traditional vaccines use a weakened or dead virus or bacteria. In RNA-based vaccines, rather than introducing a weakened or dead coronavirus the vaccine uses the strands of messenger RNA (mRNA) that will act as a platform for the production of numerous copies of a recognizable virus spike or S protein that is located on the surface of the SARS-CoV-2 virus. This S protein is very important for viral infection and immunogenicity and acts as an antigen. Once the mRNA enters into the body, the production of many spike proteins activates the immune system and triggers immunogenicity, which mounts a defense against the virus and develops antibodies to protect from future infection [23]. Currently, there are two authorized vaccines for COVID-19 that utilize lipid nanoparticles, the mRNA-1273 and the BNT162b2 vaccines. These take advantage of ionizable lipid nanoparticles to deliver nucleoside-modified mRNA which has the full-length spike protein of SARS-CoV-2. These vaccines were effective, and other vaccines are currently in clinical trials [24]. As vaccine platforms, mRNA molecules are safer and more potent than the other options.

### 2.2. Recombinant Protein Based COVID Vaccines

Since the spike S glycoprotein of the SARS-CoV virus plays an important role in virus attachment, entry and induction of neutralizing antibodies, as discussed above, the S protein is widely considered as a target for vaccine development. The recombinant protein vaccine uses a part of the whole protein or a protein fragment with a carrier protein as the antigen [25]. Once taken by the antigen-presenting cells (APC) the antigen protein is digested in the endosome, while a small fraction of the digested fragments is trimmed and presented to the major histocompatibility complex (MHC) II molecules, triggering downstream immune responses. For SARS-CoV, it was shown that animals immunized with recombinant protein vaccine candidates produced neutralizing antibodies [25,26]. Although very safe as a vaccine, the main disadvantage of the recombinant protein is that it usually only induces specific humoral immune responses and sometimes only provides partial protection to viral infections [27,28]. However, this problem can be overcome by using an adjuvant. Therefore, recombinant protein vaccines often require an adjuvant in their formulation to increase immunogenicity. For example, vaccine candidate NVX-CoV2373 for COVID-19 uses Matrix-M as the adjuvant [29]. 

### 2.3. Viral Vector Based COVID Vaccines

Viral vector-based vaccines are also common platforms for many vaccine producers. In viral vector-based vaccines, the antigen is cloned into a viral vector that lacks the ability to reproduce; therefore, it is safe to use as an antigen. Also, a single dose of vaccine is often enough to stimulate long-term protection. Some common vectors used for vaccine preparation include lentivirus, adenovirus, and adeno-associated virus (AAV). The viral vector imitates the viral infection disease state and therefore can produce stronger cellular immune responses as compared to the recombinant subunit protein vaccine. A SARS-CoV vaccine candidate using the AAV vector was investigated by Du et al. [30]. This investigation showed some breakthrough findings; the intranasal vaccination induced a systemic humoral immune response with (1) comparable strength and shorter duration than the IM; (2) stronger systemic and local-specific cytotoxic T cell responses than the intramuscular vaccination, as evidenced by higher prevalence of IL-2 and/or IFN-γ-producing CD3+/CD8+ T cells in both lungs and spleen; (3) similar protection against SARS-CoV challenge in mice as compared with intramuscular vaccination; (4) higher titers of mucosal IgA and serum-neutralizing Ab, associated with lower viral load and less pulmonary pathological damage, while no Ab-mediated disease enhancement effect was observed [31]. One high-profile example of such viral vector vaccine is the University of Oxford/AstraZeneca vaccine AZD1222 (formerly known as ChAdOx1) [32]. Two other adenovirus based COVID-19 vaccines have been approved for early or limited use internationally. One of these is called Ad5-nCoV, developed by the Chinese Academy of Military Medical Sciences with CanSino Biologics [33]. The other is Sputnik V, or Gam-COVID-Vac, developed by the Gamaleya Research Institute, part of Russia’s health ministry [34].

### 2.4. Plasmid DNA-Based COVID Vaccines

DNA vaccines eliminate the need for using live viruses, and hence have a better safety profile. The manufacturing process of plasmid DNA is relatively straightforward, and the double-stranded DNA molecules are more stable than virus, protein, and mRNA and can be freeze-dried for long-term storage. The main prohibitory factor for plasmid DNA vaccines is their low transfection efficacy, necessitating transfection modalities. For example, Inovio’s COVID-19 vaccine candidate, INO-4800, uses a handheld electroporation device, CELLECTRA. The vaccine is injected intradermally along with electrodes, then an electric pulse is applied to open the cell membrane, allowing the plasmid to enter a cell [35]. Using an established device allows for fast launch in clinical trials, but it also introduces additional hurdles in mass vaccination. 

The current SARS-CoV-2 vaccines as marketed products or in research phases are listed in Table 1.

## 3. Limitations of Current COVID-19 Vaccines

The current marketed vaccines for COVID-19 have several limitations, including side effects, preservation, transportation, distribution, route of administration, needle-fear, and anti-vaccine mentality. All these limitations are vaccine-dependent and are discussed below. 

### 3.1. Side Effects

According to the CDC, the most common side effects experienced after receiving the COVID-19 vaccine include pain at the injection site, redness, swelling, tiredness, headache, chills, fever, and nausea. Other effects include sensitive skin, menstrual cycle changes, and blood clotting [37,38]. Furthermore, because all of the vaccines are administered intramuscularly (IM), they are in a liquid form which needs to be preserved at low temperatures. This is a limitation in many countries with meager resources. Furthermore, the vaccine requires an expensive cold chain network for preservation, storage, and transportation, something that many resource-poor countries cannot afford. Therefore, a vaccine formulation that can be preserved, stored, and transported at room temperature would be most preferable and globally applicable. Furthermore, the IM route of administration involves needles, which many patients have a fear of. The anxiety associated with a needle stick and the aftermath of injection consequences result in hesitation among these patients. Thus, a needle-free delivery system can increase the mass vaccination rate. 

### 3.2. Refusal of Vaccines

One major challenge for mass vaccination is the refusal to be vaccinated. The frequency of vaccine refusal, which is associated with many factors, is increasing worldwide. However, the refusal tendency among the population varies from country to country. A study was conducted by Meriggi, N.F. et al. to analyze COVID-19 vaccine acceptance across 15 survey samples covering 10 low- and middle-income countries (LMICs) in Asia, Africa and South America, Russia (an upper-middle-income country) and the United States, for a total of 44,260 individuals [39]. The results showed considerably higher willingness to take a COVID-19 vaccine in LMIC samples (mean 80.3%; median 78%; range 30.1 percentage points) compared with the United States (mean 64.6%) and Russia (mean 30.4%). It was found that the higher vaccine acceptance in LMICs is primarily due to individuals’ interests in personal protection against COVID-19, while concern about side effects is the most common reason for hesitancy [39]. 

A study was conducted by Yigit et al. to determine the number of people that refused vaccination with COVID-19 vaccines both domestic and foreign and to identify the underlying factors for refusal [40]. Of the study participants, 63.6% were women and 36.4% were men. In terms of educational status, 12.9% were primary school graduates, 24.3% were high-school graduates, 53.7% were university graduates, 6.3% had a master’s degree, and 1.9% had a terminal degree. The research results showed that while 66.1% of patients were reluctant to receive foreign COVID-19 vaccines, only 37.4% were reluctant to receive domestic COVID-19 vaccines. The most common reasons for refusal were anxiety about vaccine side effects, lack of knowledge about the effectiveness of vaccines, and distrust of vaccines originating from abroad [40]. 

### 3.3. Blood Clotting

The blood clotting effect after COVID vaccination has recently received attention from scientists and media. Blood clotting has mostly occurred as a result of the COVID vaccine produced by Johnson and Johnson. Several cases of blood clotting after vaccination have been reported [41]. As of 12 April 2021, more than 6.8 million doses of the Johnson & Johnson (Janssen) vaccine had been administered in the U.S. The CDC and FDA reviewed the data involving six reported U.S. cases of a rare and severe type of blood clot in individuals after receiving the J&J vaccine. In these cases, a type of blood clot called cerebral venous sinus thrombosis (CVST) was seen in combination with low levels of blood platelets (thrombocytopenia). Injections of Johnson & Johnson’s coronavirus vaccine were halted across the USA on 13 April 2021, after federal health agencies called for a pause in the vaccine’s use as they examined the rare blood-clotting disorder that emerged in six recipients. Besides the USA, Johnson & Johnson also delayed the rollout of its vaccine in Europe, where several countries were poised to start administering the vaccine at around the same time. Additionally, South Africa, which was devastated by a more contagious variant of the virus that had emerged there, suspended use of the vaccine. Australia also announced it would not purchase any Johnson & Johnson vaccine [42]. 

### 3.4. Needle Fear

A study was conducted to evaluate the prevalence of needle fear and summarize the characteristics of individuals who exhibit this fear [43]. Most children exhibited needle fear, while prevalence estimates for needle fear ranged from 20–50% in adolescents and 20–30% in young adults. In general, needle fear decreased with increasing age. Both needle fear and needle phobia were more prevalent in females than males. Avoidance of influenza vaccination because of needle fear occurred in 16% of adult patients, 27% of hospital employees, 18% of workers at long-term care facilities, and 8% of healthcare workers at hospitals. Needle fear was common when undergoing venipunctures and blood donations, and in those with chronic conditions requiring injection [43]. This fear of needles can result in avoidance of preventative measures and treatment of various diseases, including COVID-19. Currently, all the COVID vaccines are IM injectable. As such, needle fear has hindered mass vaccination thus far during the pandemic. 

### 3.5. Route of Administration

Route of administration plays an important role in vaccination outcomes, as it can affect the extent and quality of immune response [44,45]. Almost all the current COVID vaccines are designed for intramuscular administration. Since COVID-19 primarily causes respiratory infection, developing mucosal immune protection is critical as it provides additional protection. Thus, mucosal vaccinations (e.g., intranasal, pulmonary, oral) might be superior to parenteral vaccinations. 

Most of these limitations can be addressed by making a particulate vaccine formulation using nanoparticles or microparticles. Particulate formulation can be administered by oral, sublingual, or buccal route. Also, the particulate vaccines can be easily prepared at large scale, and particulate formulations can be preserved, stored, and transported at room temperature [17]. 

## 4. Potential of Micro- or Nano Particulate COVID Vaccines

A particulate COVID vaccine can be produced by loading an antigen or drug of interest into nanoparticles or microparticles. These particles can be administered via the oral, sublingual, or buccal route. Particulate vaccines have been developed and studied before. The measles vaccine’s microparticles were made with biocompatible and biodegradable bovine serum albumin (BSA) polymer and processed by spray-dried production of microparticles. These vaccine microparticles were then incorporated into an orally dissolvable film. The vaccine particles were non-cytotoxic, induced a significant innate immune response, and increased the antigen presentation and co-stimulatory molecule expression of antigen-presenting cells. In vivo, the ODF vaccine formulation was tested in juvenile pigs. After 2 weeks, there was a significantly higher antibody titer plateauing through week 6. The results from this study suggest that the ODF measles vaccine formulation is a viable alternative dosage form for noninvasive immunization [46]. 

A particulate vaccine formulation has huge potential, as the particle can possibly be used as antigen carrier and an adjuvant. Particulate carriers can serve as effective antigen delivery systems that are able to enhance and/or facilitate the uptake of antigens by antigen-presenting cells (APCs) such as dendritic cells (DCs) or macrophages [47,48]. Furthermore, when delivered orally, particulate vaccine formulations have the ability to protect the integrity of antigens against acidic and enzymatic degradation in the stomach and GI tract until they are delivered to the immune cells [49,50]. 

Another advantage of using a particulate vaccine formulation is that it can eliminate the use of adjuvants which have minimal immunogenic effect. The immunologic effect of particulate vaccines is related to the size, stability, antigen-loading and antigen-release kinetic properties of the particle [51]. The immune response is also influenced by particle interaction with APCs and antigen presentation and processing by APCs [52]. 

Micro- or nanoparticles have some unique physiochemical properties that make them ideal candidates for vaccine delivery. They have a higher surface-to-volume ratio, small size, the ability to encapsulate various drugs, and tunable surface chemistry, all of which gives them many advantages over their bulky counterparts. These advantages include multivalent surface modification with targeting ligands, efficient navigation of the complex in an in vivo environment, enhanced intracellular trafficking, and the potential for addition of charged particles to increase target selectivity and sustained release of drug [17]. These advantages make nanoparticles ideal candidates for formulating vaccine delivery systems that can be applied for COVID vaccines. 

The advantages of nanoparticle-based delivery of vaccines and drugs include improved biological stability of the antigen or drug and efficacy in targeting APCs for induction of innate and adaptive immunity due to Class I and Class II presentations [53]. Nanoparticles may also provide enhanced intracellular concentrations, controlled release of vaccine antigen or drug, and a reduced number of administrations due to enhanced immune response. Furthermore, nano-sized particles can themselves act as immune stimulating adjuvants. Gamvrellis et al. have shown that a nano-particulate antigen delivery system was able to induce a substantial immune response without inducing any inflammation [54]. 

A nanoparticle formulation of a vaccine is more immunogenic when compared to the solution form of the antigen. It has been found that poly (d, l-lactic-co-glycolic acid) nanoparticles (PLGA-NPs) can be used to formulate a vaccine delivery system which has potential in the development of future therapeutic cancer vaccines [55]. This nanoparticle can target dendritic cells (DCs) which can effectively initiate antitumor activity. The PLGA nanoparticle-containing antigens along with immune-stimulatory molecules (adjuvants) can target not only DCs but also provide immune activation and rescue impaired DCs from tumor-induced immunosuppression [20]. The authors further assessed the extent of maturation of DCs after treatment with the antigen, monophosphoryl lipid A (MPLA), and encapsulated PLGA nanoparticles. The generation of primary T-cell immune responses elicited by DCs was monitored. Results showed that the high amounts of pro-inflammatory and TH1 (T helper 1) polarizing cytokines and chemokines released by the nanoparticles are greater than that achieved by MPLA in solution [56]. 

Biodegradable and biocompatible polymers, copolymers and lipids can be used for COVID particulate vaccine preparation. It has been found previously that these polymers have been used to prepare nano/micro-particles as vaccine-delivery systems [54,57,58]. The material is selected based on several factors, including biocompatibility, degradation rate, hydrophilicity or lipophilicity, surface charge, and polarity. The SARS-2 virus infects mainly the area of the lungs, therefore nanoparticulate formulation has the advantage in fighting this virus due to their ability to reach deeper into the lung area.

**Figure 3:**
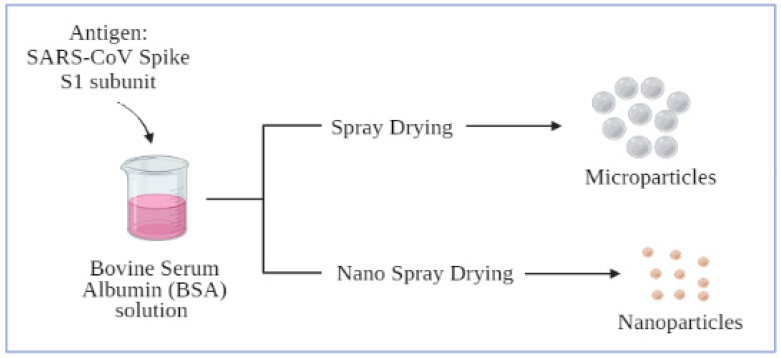
Formulation of microparticles or nanoparticles using spray drying method.

Figure 3 shows how the spray drying method can be utilized for producing nano- or microparticles [59,60]. A biodegradable polymer-based particulate vaccine can act as an adjuvant itself. Therefore, there may be no need for using salt-based adjuvants, thus, eliminating the adverse effects caused by adjuvants. In addition, it is also possible to increase the efficiency of the particulate vaccine by adding appropriate ligands, charged particles or any other biocompatible chemicals to increase the specificity of the nano- or microparticles for targeted delivery [61]. 

## 5. Possible Particulate COVID Vaccine Delivery System for Oral, Sublingual and Buccal Administrations

### 5.1. Oral Administration

Oral administration is the most preferred route for drug delivery as it is the most patient compliant. Oral administration of vaccines is more acceptable to the patient as it is needle-free, easy to administer, requires less-trained personnel for administration, and is easy to apply to the mass population. One of the most common oral vaccines is the oral polio vaccine, which has been used to eradicate polio. The oral polio vaccine has been the mainstay of the global polio eradication initiative (GPEI) in most countries. The polio vaccine allows for the encounter of the polio virus by the immune system to be less threatening while still allowing the body to mount a humoral response for protection against any future exposure to the virus [62]. Upon administration, the vaccine elicits a local immune response in the intestinal mucous membranes, a location at which the poliovirus multiplies [62,63]. After administration, the live-attenuated oral poliovirus vaccine replicates in gut-associated tissues, eliciting mucosal and systemic immunity. The oral polio vaccine is both therapeutic and preventative, protects from disease, and limits poliovirus spread. As such, mass vaccination with the oral polio vaccine has been used as a strategy to end the circulation of all polioviruses [64].

Acknowledging this, developing an oral COVID vaccine with high efficiency and low cost will eliminate the limitations that the current vaccines have. The oral route will also eliminate the need for trained personnel to administer the vaccine, which will give the vaccine a more global character as it will be easily available and applicable in resource-poor countries. In addition, oral vaccines have the potential to stimulate mucosa-associated lymphoid tissue (MALT) located in the digestive tract and gut-associated lymphoid tissue (GALT). About half the lymphocytes of the immune system are in the MALT [65]. Structurally, MALT tissue ranges from loose, barely organized clusters of lymphoid cells in lamina propria of intestinal villi to well-organized structures such as tonsils, appendix and Peyer’s patches [66]. The tonsils are found in three locations: lingual at the base of the tongue, palatine at the sides of the back of the mouth, and pharyngeal, in the adenoids. Also, under the epithelial cell layer of lamina propria and tonsils, there are many B cells, plasma cells, activated TH cells, and macrophages [66]. MALT can be functionally divided into effector and inductive sites [65]. Inductive sites contain secondary lymphoid tissues in which IgA class switching and clonal expansion of B-cells occurs in response to antigen specific T-cell activation. After activation and IgA class switching, T- and B-cells migrate from inductive sites to effector sites. Effector sites are present in all mucosal tissues as disseminated lymphoid tissues diffusely distributed throughout the lamina or substantia propria [65]. In effector sites, secretory IgA, or S-IgA (two IgA molecules joined by a J-chain and bound to a secretory component, an epithelial cell membrane receptor) is secreted across the mucosal epithelium [67]. Therefore, oral administration, and even sublingual and buccal administration, takes advantage of this location and structure of MALT tissues. For the SARS-2 virus, an elevated mucosal immune response could serve as a first line of protection against infection. The oral delivery of vaccines as an alternative immunization route and the efficiency of mucosal immunization for different antigens has been studied [68]. In addition, the intranasal route of administration for vaccine delivery has been investigated. Results from studies of both oral and intranasal routes of administration show the potential of mucosal immunization with VLP-based HPV vaccines [68,69]. A COVID vaccine which is mRNA-based is in particulate form with lipids but is injected as a solution via the IM route. Therefore, the vaccine is already in particulate form, which can be converted into microparticles by adding other polymers, and these microparticles can be delivered via the oral route in solution or suspension form. 

Also, when dosed intranasally, a vaccine is intrinsically prone to inducing Th17 immune responses [31], which may not be ideal for the clearance of SARS-CoV-2 viral particles from the lungs. Another limiting factor for a nasal or pulmonary COVID-19 vaccine is the need for a special and costly delivery device, which may also exert pressure on the vaccine formulation. For example, a loss of virus titer was observed when using a nebulizer to deliver a live virus formulation [70]. Therefore, it is not surprising that almost all COVID-19 vaccine candidates advanced to clinical trials are given by injection (see Table 1), although they may not induce specific mucosal immunity [35]. 

### 5.2. Film-Based Particulate COVID Vaccine for Sublingual and Buccal Administration

**Figure 4:**
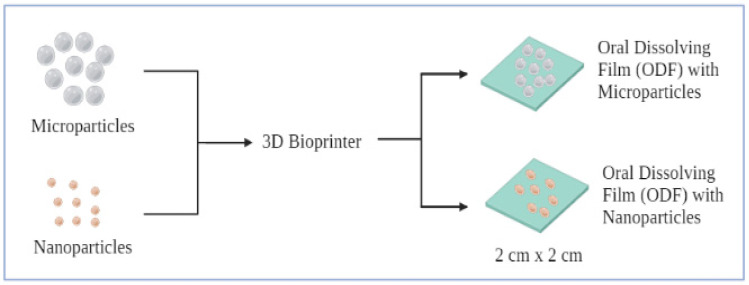
Formulation of oral dissolving films (ODF) with either microparticles or nanoparticles using 3D bioprinter.

Although placed in the oral cavity, the administration of drugs via sublingual and buccal routes are different from oral (per oral, PO) administration. Unlike oral routes, sublingual, or buccal routes are systemic, directly accessible to the blood. The nano- or microparticle of vaccine-loaded films can be prepared by solvent casting or using a 3D bioprinter (Figure 4) [71]. Instead of passing through the GI tracts such as the esophagus, stomach or intestine, the drug can directly enter the blood through the membrane. Medications taken by buccal or sublingual administration provide consistent drug concentration levels in the blood, dissolve quickly, have immediate onset of action, and can avoid the first pass effect. Since there is no first pass effect, the bioavailability is high. Therefore, compared to oral administration, less drug can be used to elicit the desired effect. Additionally, the patient does not need to swallow the drug in sublingual or buccal administration. Another advantage of buccal and sublingual administration is that they do not subject proteins and/or peptides to the degradation that is usually caused by gastrointestinal administration [72]. Also, oral films are easy to prepare, administer, and handle. Normally, any biodegradable and biocompatible polymers can be used to prepare the film, including any other material needed such as a permeation enhancer, plasticizer, etc., in a simple method [72]. The most important advantage of buccal and sublingual administration is that the vaccine can produce both systemic and mucosal immunity [73]. SARS-2 virus infects the host through the mucosa. Several signs after COVID infection, such as loss of taste, dry mouth, and mucosal lesions such as ulcerations, enanthema, and macules imply that the virus infects the mucosa. However, the mucosal infection has not been completely understood. To address this, Sinjari B et al. and Huang et al. have generated and analyzed two single-cell RNA sequencing datasets of the human minor salivary glands and gingiva. Their studies showed that the oral cavity is an important site for SARS-CoV-2 infection and implicates saliva as a potential route of SARS-CoV-2 transmission [74,75]. Therefore, an ideal COVID vaccine should induce protective immunity at mucosal sites to act as a first line of defense against infections. However, most of the vaccines currently in use are administered via injection (such as intramuscular route) and have very limited mucosal immunity. However, vaccines administered via mucosal routes have proven to be effective for the induction of both systemic and local immunity [76]. Additionally, mucosal immunization via sublingual and buccal administration makes vaccine delivery easier and safer than parenteral administration routes. These are very suitable for mass immunizations during pandemic situations and improve vaccine acceptability, especially among children [77]. Therefore, mucosal administration of vaccines via buccal or sublingual routes could be a great choice for mass protection. Among the two, buccal drug delivery was identified as a better option for administration. A quickly-soluble tablet or film dosage form can be used as drug carrier for buccal administration. The quickly-soluble oral film dosage form has several advantages over other dosage forms for vaccines or drugs. Lower bioavailability of solid oral drugs, the inconvenience of administering injections, and inaccurate dosing by liquid formulations have turned the focus of pharmaceutical companies to developing oral film forms of medications that eliminate several of these limitations. Oral films are easy to prepare, administer, and handle. Normally, any biodegradable and biocompatible polymer can be used to prepare the film, including any other material needed such as permeation enhancer, plasticizer, etc., by a simple method.

## 6. Conclusions

The emergence of the highly pathogenic coronavirus SARS-CoV-2 throughout the world in 2020 has posed a serious threat to global health. As of today, the pandemic is not yet fully controlled, in fact, some countries are having a second or third wave which is more dangerous than the first one. There are already six vaccines that have been marketed and more are in the pipeline worldwide. However, due to the limitations of the current vaccines, the goal of global COVID-19 vaccination has not been reached. The limitations of the current COVID vaccines can be addressed by developing a particulate vaccine drug delivery system to be administered via oral, sublingual, or buccal routes. A specific antigen of interest can be incorporated into a nanoparticle or microparticle formulation that can be delivered via a vaccine. The particulate forms also have several advantages over the solution forms; they are easy to prepare and scale up, can be kept at room temperature, and can elicit mucosal along with systemic immunity. Thus, it is expected that particulate formulation of COVID vaccines may help to enhance the vaccination program globally. 

## Figures and Tables

**Table 1:** List of some of the current marketed and investigational vaccine candidates for SARS-CoV-2.

Vaccine Candidates	Classification of Vaccine	Clinical Phase	Lead Development Company/Collaboration
GRAd-COV2	Adenovirus-based vaccine	Phase 1	ReiThera; Leukocare; Univercells; Lazzaro Spallanzani National Institute for Infection
ChAd-SARS-CoV-2-S	Adenovirus-based vaccine	Preclinical	Washington University School of Medicine in St. Louis
LinealDNA	DNA Vaccine	Preclinical	Takis Biotech
AG0301-COVID19	DNA vaccine	Phase 1/2	AnGes, Inc.
GX-19	DNA vaccine	Phase 1/2	Genexine
INO-4800	DNA vaccine (plasmid)	Phase 1/2	Inovio Pharmaceuticals; Center for Pharmaceutical Research, Kansas City. Mo.; University of Pennsylvania, Philadelphia
ZyCoV-D	DNA vaccine (plasmid)	Phase 2	Zydus Cadila
AAVCOVID	Gene-based vaccine	Preclinical	Massachusetts General Hospital; University of Pennsylvania
No name given	gp96-based vaccine	Preclinical	Heat Biologics; University of Miami Miller School of Medicine
No name given	Ii-Key peptide COVID-19 vaccine	Preclinical	Generex Biotechnology
No name given	Inactivated vaccine	Phase 1/2	Research Institute for Biological Safety Problems, Rep of Kazakhstan
No name given	Inactivated vaccine	Phase 3	Wuhan Institute of Biological Products; China National Pharmaceutical Group (Sinopharm); Henan Provincial Center for Disease Control and Prevention
Covaxin	Inactivated vaccine	Phase 2	Bharat Biotech; National Institute of Virology
No name given	Inactivated vaccine	Phase 1/2	Chinese Academy of Medical Sciences, Institute of Medical Biology; West China Second University Hospital, Yunnan Center for Disease Control and Prevention
No name given	Inactivated vaccine	Preclinical	Shenzhen Kangtai Biological Products
BBIBP-CorV	Inactivated vaccine	Phase 3	Beijing Institute of Biological Products; China National Pharmaceutical Group (Sinopharm); Henan Provincial Center for Disease Control and Prevention
CoronaVac	Inactivated vaccine (formalin with alum adjuvant)	Phase 3	Sinovac; Sinovac Research and Development Co., Ltd.
AdCOVID	Intranasal vaccine	Preclinical	Altimmune; University of Alabama at Birmingham
T-COVIDTM	Intranasal vaccine	Preclinical	Altimmune
Bacillus Calmette-Guerin (BCG) vaccine	Live-attenuated vaccine	Phase 2/3	University of Melbourne and Murdoch Children’s Research Institute; Radboud University Medical Center; Faustman Lab at Massachusetts General Hospital
V591	Replicating viral vector	Phase 1	University of Pittsburgh’s Center for Vaccine Research; Themis Biosciences; Institut Pasteur
bacTRL-Spike	Monovalent oral vaccine (bifidobacteria)	Preclinical	Symvivo
COVAX-19	Protein subunit Monovalent spike protein vaccine	Phase 1	Vaxine Pty Ltd.; Royal Adelaide Hospital
No name announced	mRNA lipid nanoparticle	Early research	CanSino Biologics, Precision NanoSystems
mRNA-1273	LNP-encapsulated mRNA vaccine	Phase 3	Moderna; Kaiser Permanente Washington Health Research Institute
BNT162	3 LNP-mRNAs-based vaccine	Phase 3	Pfizer, BioNTech
CVnCoV	mRNA-based vaccine	Phase 1	CureVac
No name given	mRNA-based vaccine	Preclinical	Chulalongkorn University’s Center of Excellence in Vaccine Research and Development
UB-612	Multitope peptide-based vaccine	Phase 1	COVAXX; United Biomedical Inc. (UBI)
JNJ-78436735	Non-replicating viral vector	Phase 3	Johnson & Johnson
Sputnik V COVID-Vac; Гам-КОВИД-Вак^20^	Non-replicating viral vector Adenovirus-based vaccine	Phase 3	Gamaleya Research Institute, Acellena Contract Drug Research and Development
EpiVacCorona	Peptide vaccine	Phase 1/2	Federal Budgetary Research Institution State Research Center of Virology and Biotechnology
No name given	Plant-based adjuvant vaccine	Phase 1	Medicago; GSK; Dynavax
No name given	Protein subunit vaccine S protein^20^	Phase 1/2	Sanofi; GlaxoSmithKline
No name given	Protein subunit RBD-based vaccine	Phase 1/2	Kentucky Bioprocessing, Inc.
AdimrSC-2f	Protein subunit vaccine	Phase 1	Adimmune
No name given	Protein subunit vaccine	Phase 1	CSL; The University of Queensland; Seqirus
SCB-2019	Protein subunit vaccine	Phase 1	GlaxoSmithKline, Sanofi, Clover Biopharmaceuticals, Dynavax and Xiamen Innovax; Linear Clinical Research (Australia)
No name given	Protein subunit vaccine	Preclinical	University of Saskatchewan Vaccine and Infectious Disease Organization-International Vaccine Centre
PittCoVacc	Recombinant protein subunit vaccine	Preclinical	UPMC/University of Pittsburgh School of Medicine
RBD-Dimer (relief)	Protein subunit Adjuvanted recombinant protein	Phase 2	Anhui Zhifei Longcom Biopharmaceutical, Institute of Microbiology of the Chinese Academy of Sciences
No name given	Recombinant vaccine	Preclinical	Sanofi, Translate Bio
Ad5-nCoV	Recombinant vaccine (adenovirus type 5 vector)	Phase 3	CanSino Biologics; Tongji Hospital
VXA-CoV2-1	Recombinant vaccine (adenovirus vector)	Phase 1	Vaxart
V590	Recombinant vaccine (stomatitis virus)	Phase 1	Merck; IAVI
DelNS1-2019-nCoV-RBD-OPT1	Replicating viral vector	Phase 1	Xiamen University, Beijing Wantai Biological Pharmacy; Jiangsu Provincial Centre for Disease Control and Prevention
No name announced	Replicating viral vector	Preclinical	Federal Budgetary Research Institution (FBRI); State research center of virology and biotechnology “VECTOR”
AZD1222 ChAdOx1-S^20^	Non-Replicating viral vector vaccine	Phase 3	The University of Oxford; AstraZeneca; IQVIA; Serum Institute of India
HDT-301	RNA vaccine	Preclinical	University of Washington; National Institutes of Health Rocky Mountain Laboratories; HDT Bio Corp.
LNP-CoVsaRNA	Self-amplifying RNA vaccine	Phase 1/2	Imperial College London
HaloVax	Self-assembling vaccine	Preclinical	Voltron Therapeutics, Inc.; Hoth Therapeutics, Inc.; MGH Vaccine and Immunotherapy Center
LUNAR-COV19 [36]	Self-replicating RNA vaccine	Phase 1/2	Arcturus Therapeutics and Duke-NUS Medical School
CDX-005 [10,12]	Weakened	Phase 1	Codagenix, Serum Institute of India

## Data Availability

Data are available in correspondent journal.

## References

[1] Kahn J.S., McIntosh K. (2005). Discussion. Pediatr. Infect. Dis. J..

[2] McIntosh K., Dees J.H., Becker W.B., Kapikian A.Z., Chanock R.M. (1967). Recovery in Tracheal Organ Cultures of Novel Viruses from Patients with Respiratory Disease. Proc. Natl. Acad. Sci. USA.

[3] Tyrrel D.A.J., Lm Eida J.D.A., Akstelskaya L.Z., Easterday B.C., Bingham R.W. (1975). Coronaviridae1. Intervirology.

[4] First Travel-Related Case of 2019 Novel Coronavirus Detected in United States|CDC Online Newsroom|CDC. https://www.cdc.gov/media/releases/2020/p0121-novel-coronavirus-travel-case.html.

[5] Second Travel-Related Case of 2019 Novel Coronavirus Detected in United States|CDC Online Newsroom|CDC. https://www.cdc.gov/media/releases/2020/p0124-s-travel-coronavirus.html.

[6] New-Type Coronavirus Causes Pneumonia in Wuhan: Expert-Xinhua|English.news.cn. http://www.xinhuanet.com/english/2020-01/09/c_138690570.htm.

[7] Coronavirus|Human Coronavirus Types|CDC. https://www.cdc.gov/coronavirus/types.html.

[8] Coronavirus (COVID-19) Frequently Asked Questions | CDC. https://www.cdc.gov/coronavirus/2019-ncov/faq.html.

[9] Kuba K., Imai Y., Rao S., Gao H., Guo F., Guan B., Huan Y., Yang P., Zhang Y., Deng W. (2005). A crucial role of angiotensin converting enzyme 2 (ACE2) in SARS coronavirus-induced lung injury. Nat. Med. Vol..

[10] Jeffers S.A., Tusell S.M., Gillim-Ross L., Hemmila E.M., Achenbach J.E., Babcock G.J., Thomas W.D., Thackray L.B., Young M.D., Mason R.J. (2004). Cd209l (L-Sign) Is a Receptor for Severe Acute Respiratory Syndrome Coronavirus. Proc. Natl. Acad. Sci. USA.

[11] Belouzard S., Millet J.K., Licitra B.N., Whittaker G.R. (2012). Mechanisms of Coronavirus Cell Entry Mediated by the Viral Spike Protein. Viruses.

[12] Callaway E. (2020). The Race for coronavirus vaccines. Nature.

[13] Knight S.C., Stagg A.J. (1993). Antigen-presenting cell types. Curr. Opin. Immunol..

[14] Levi R., Azzolini E., Pozzi C., Ubaldi L., Lagioia M., Mantovani A., Rescigno M. (2021). One dose of SARS-CoV-2 vaccine exponentially increases antibodies in individuals who have recovered from symptomatic COVID-19. J. Clin. Investig..

[15] Ledford H. (2021). COVID vaccines and blood clots: Five key questions. Nature.

[16] Greinacher A., Thiele T., Warkentin T.E., Weisser K., Kyrle P.A., Eichinger S. (2021). Thrombotic Thrombocytopenia after ChAdOx1 nCov-19 Vaccination. N. Engl. J. Med..

[17] Uddin M.N., Kouzi S.A., Hussain M.D. (2015). Strategies for Developing Oral Vaccines for Human Papillomavirus (HPV) Induced Cancer Using Nanoparticle Mediated Delivery System. J. Pharm. Pharm. Sci..

[18] Copland M.J., Baird M.A., Rades T., McKenzie J.L., Becker B., Reck F., Tyler P.C., Davies N.M. (2003). Liposomal delivery of antigen to human dendritic cells. Vaccine.

[19] Schijns V.E. (2000). Immunological concepts of vaccine adjuvant activity. Curr. Opin. Immunol..

[20] Kreuter J. (1996). Nanoparticles and microparticles for drug and vaccine delivery. J. Anat..

[21] Parums D.V. (2021). Editorial: SARS-CoV-2 mRNA Vaccines and the Possible Mechanism of Vaccine-Induced Immune Thrombotic Thrombocytopenia (VITT). Med. Sci. Monit..

[22] Verga D. mRNA and the Future of Vaccine Manufacturing | PATH. https://www.path.org/articles/mrna-and-future-vaccine-manufacturing/.

[23] Florindo H.F., Kleiner R., Vaskovich-Koubi D., Acúrcio R.C., Carreira B., Yeini E., Tiram G., Liubomirski Y., Satchi-Fainaro R. (2020). Immune-mediated approaches against COVID-19. Nat. Nanotechnol..

[24] Hou X., Zaks T., Langer R., Dong Y. (2021). Lipid nanoparticles for mRNA delivery. Nat. Rev. Mater..

[25] He Y., Zhou Y., Liu S., Kou Z., Li W., Farzan M., Jiang S. (2004). Receptor-binding domain of SARS-CoV spike protein induces highly potent neutralizing antibodies: Implication for developing subunit vaccine. Biochem. Biophys. Res. Commun..

[26] Du L., Zhao G., He Y., Guo Y., Zheng B.-J., Jiang S., Zhou Y. (2007). Receptor-binding domain of SARS-CoV spike protein induces long-term protective immunity in an animal model. Vaccine.

[27] Fang M., Cheng H., Dai Z., Bu Z., Sigal L.J. (2006). Immunization with a single extracellular enveloped virus protein produced in bacteria provides partial protection from a lethal orthopoxvirus infection in a natural host. Virology.

[28] Galmiche M.C., Goenaga J., Wittek R., Rindisbacher L. (1999). Neutralizing and Protective Antibodies Directed against Vaccinia Virus Envelope Antigens. Virology.

[29] Novavax to Present COVID-19 Vaccine Candidate Progress in World Vaccine Congress Webinar Series | Novavax Inc.—IR Site. https://ir.novavax.com/news-releases/news-release-details/novavax-present-covid-19-vaccine-candidate-progress-world.

[30] Thi T.T.H., Suys E.J.A., Lee J.S., Nguyen D.H., Park K.D., Truong N.P. (2021). Lipid-Based Nanoparticles in the Clinic and Clinical Trials: From Cancer Nanomedicine to COVID-19 Vaccines. Vaccines.

[31] Du L., Zhao G., Lin Y., Sui H., Chan C., Ma S., He Y., Jiang S., Wu C., Yuen K.-Y. (2008). Intranasal Vaccination of Recombinant Adeno-Associated Virus Encoding Receptor-Binding Domain of Severe Acute Respiratory Syndrome Coronavirus (SARS-CoV) Spike Protein Induces Strong Mucosal Immune Responses and Provides Long-Term Protection against SARS-CoV infection. J. Immunol..

[32] Voysey M., Clemens S.A.C., Madhi S.A., Weckx L.Y., Folegatti P.M., Aley P.K., Angus B., Baillie V.L., Barnabas S.L., Bhorat Q.E. (2021). Safety and efficacy of the ChAdOx1 nCoV-19 vaccine (AZD1222) against SARS-CoV-2: An interim analysis of four randomised controlled trials in Brazil, South Africa, and the UK. Lancet.

[33] Lewis D. (2020). China’s coronavirus vaccine shows military’s growing role in medical research. Nature.

[34] Cohen J. (2020). Russia’s approval of a COVID-19 vaccine is less than meets the press release. Science.

[35] Wang J., Peng Y., Xu H., Cui Z., Williams R.O. (2020). The COVID-19 Vaccine Race: Challenges and Opportunities in Vaccine Formulation. AAPS PharmSciTech.

[36] Uddin M., Henry B., Carter K.D., Roni M.A., Kouzi S.A. (2019). A Novel Formulation Strategy to Deliver Combined DNA and VLP Based HPV Vaccine. J. Pharm. Pharm. Sci..

[37] Blumenthal K.G., Freeman E.E., Saff R.R., Robinson L.B., Wolfson A.R., Foreman R.K., Hashimoto D., Banerji A., Li L., Anvari S. (2021). Delayed Large Local Reactions to mRNA-1273 Vaccine against SARS-CoV-2. N. Engl. J. Med..

[38] Pfizer vs. Moderna Vaccines: Does One Have More Side Effects?—NBC Chicago. https://www.nbcchicago.com/news/local/pfizer-vs-moderna-vaccines-does-one-have-more-side-effects-than-the-other/2499425/.

[39] Solís Arce J.S., Warren S.S., Meriggi N.F., Scacco A., McMurry N., Voors M., Syunyaev G., Malik A.A., Aboutajdine S., Adeojo O. (2021). COVID-19 vaccine acceptance and hesitancy in low- and middle-income countries. Nat. Med..

[40] Yigit M., Ozkaya-Parlakay A., Senel E. (2021). Evaluation of COVID-19 Vaccine Refusal in Parents. Pediatr. Infect. Dis. J..

[41] Joint CDC and FDA Statement on Johnson & Johnson COVID-19 Vaccine | CDC Online Newsroom | CDC. https://www.cdc.gov/media/releases/2021/s0413-JJ-vaccine.html.

[42] Johnson & Johnson Delays Its COVID-19 Vaccine Rollout in EUROPE—The New York Times. https://www.nytimes.com/2021/04/13/world/johnson-covid-vaccine-europe.html.

[43] McLenon J., Rogers M.A.M. (2019). The fear of needles: A systematic review and meta-analysis. J. Adv. Nurs..

[44] Belyakov I.M., Ahlers J.D. (2009). What Role Does the Route of Immunization Play in the Generation of Protective Immunity against Mucosal Pathogens?. J. Immunol..

[45] Mohanan D., Slütter B., Henriksen-Lacey M., Jiskoot W., Bouwstra J.A., Perrie Y., Kündig T.M., Gander B., Johansen P. (2010). Administration routes affect the quality of immune responses: A cross-sectional evaluation of particulate antigen-delivery systems. J. Control. Release.

[46] Gala R.P., Popescu C., Knipp G.T., McCain R.R., Ubale R.V., Addo R., Bhowmik T., Kulczar C.D., D’Souza M.J. (2017). Physicochemical and Preclinical Evaluation of a Novel Buccal Measles Vaccine. AAPS PharmSciTech.

[47] Anderson E.J., Rouphael N.G., Widge A.T., Jackson L.A., Roberts P.C., Makhene M., Chappell J.D., Denison M.R., Stevens L.J., Pruijssers A.J. (2020). Safety and Immunogenicity of SARS-CoV-2 mRNA-1273 Vaccine in Older Adults. N. Engl. J. Med..

[48] Walter E., Dreher D., Kok M., Thiele L., Kiama S.G., Gehr P., Merkle H.P. (2001). Hydrophilic poly(dl-lactide-co-glycolide) microspheres for the delivery of DNA to human-derived macrophages and dendritic cells. J. Control. Release.

[49] Slütter B., Soema P.C., Ding Z., Verheul R., Hennink W., Jiskoot W. (2010). Conjugation of ovalbumin to trimethyl chitosan improves immunogenicity of the antigen. J. Control. Release.

[50] O’Hagan D. (1998). Microparticles and polymers for the mucosal delivery of vaccines. Adv. Drug Deliv. Rev..

[51] Panyam J., Labhasetwar V. (2003). Biodegradable nanoparticles for drug and gene delivery to cells and tissue. Adv. Drug Deliv. Rev..

[52] Tobío M., Gref R., Sánchez A., Langer R., Alonso M.J. (1998). Stealth PLA-PEG nanoparticles as protein carriers for nasal administration. Pharm. Res..

[53] Parkin J., Cohen B. (2001). An overview of the immune system. Lancet.

[54] Gamvrellis A., Gloster S., Jefferies M., Mottram P.L., Smooker P., Plebanski M., Scheerlinck J.-P.Y. (2013). Characterisation of local immune responses induced by a novel nano-particle based carrier-adjuvant in sheep. Vet. Immunol. Immunopathol..

[55] Thiele L., Merkle H.P., Walter E. (2003). Phagocytosis and phagosomal fate of surface-modified microparticles in dendritic cells and macrophages. Pharm. Res..

[56] Elamanchili P., Diwan M., Cao M., Samuel J. (2004). Characterization of poly (d,l-lactic-co-glycolic acid) based nanoparticulate system for enhanced delivery of antigens to dendritic cells. Vaccine.

[57] Hamdy S., Haddadi A., Hung R.W., Lavasanifar A. (2011). Targeting dendritic cells with nano-particulate PLGA cancer vaccine formulations. Adv. Drug. Deliv. Rev..

[58] Elamanchili P., Lutsiak C.M.E., Hamdy S., Diwan M., Samuel J. (2007). “Pathogen-Mimicking” Nanoparticles for Vaccine Delivery to Dendritic Cells. J. Immunother..

[59] Nettey H., Haswani D., Oettinger C.W., D’Souza M.J. (2006). Formulation and testing of vancomycin loaded albumin microspheres prepared by spray-drying. J. Microencapsul..

[60] Joshi D., Chbib C., Uddin M.N., D’Souza M.J. (2021). Evaluation of Microparticulate (S)-4,5-Dihydroxy-2,3-pentanedione (DPD) as a Potential Vaccine Adjuvant. AAPS J..

[61] Combadière B., Mahé B. (2008). Particle-based vaccines for transcutaneous vaccination. Comp. Immunol. Microbiol. Infect. Dis..

[62] O’Grady M., Bruner P.J. (2021). Polio Vaccine. StatPearls. http://www.ncbi.nlm.nih.gov/pubmed/30252295.

[63] Poliomyelitis. https://www.who.int/teams/health-product-policy-and-standards/standards-and-specifications/vaccines-quality/poliomyelitis.

[64] Yeh M.T., Bujaki E., Dolan P.T., Smith M., Wahid R., Konz J., Weiner A.J., Bandyopadhyay A.S., Van Damme P., De Coster I. (2020). Engineering the Live-Attenuated Polio Vaccine to Prevent Reversion to Virulence. Cell Host Microbe.

[65] Cesta M.F. (2006). Normal Structure, Function, and Histology of Mucosa-Associated Lymphoid Tissue. Toxicol. Pathol..

[66] Goldsby R., Kindt T., Osborne B., Kuby J. (2002). Immunology.

[67] Pabst R. (1987). The anatomical basis for the immune function of the gut. Anat. Embryol..

[68] Thönes N., Müller M. (2007). Oral immunization with different assembly forms of the HPV 16 major capsid protein L1 induces neutralizing antibodies and cytotoxic T-lymphocytes. Virology.

[69] Chang S., Warner J., Liang L., Fairman J. (2009). A novel vaccine adjuvant for recombinant flu antigens. Biologicals.

[70] Smith J.H., Papania M., Knaus D., Brooks P., Haas D.L., Mair R., Barry J., Tompkins S.M., Tripp R.A. (2012). Nebulized live-attenuated influenza vaccine provides protection in ferrets at a reduced dose. Vaccine.

[71] Panraksa P., Udomsom S., Rachtanapun P., Chittasupho C., Ruksiriwanich W., Jantrawut P. (2020). Hydroxypropyl Methylcellulose E15: A Hydrophilic Polymer for Fabrication of Orodispersible Film Using Syringe Extrusion 3D Printer. Polymers.

[72] Uddin M.N., Allon A., Roni M.A., Kouzi S. (2019). Overview and Future Potential of Fast Dissolving Buccal Films as Drug Delivery System for Vaccines. J. Pharm. Pharm. Sci..

[73] Anselmo A.C., Gokarn Y., Mitragotri S. (2019). Non-invasive delivery strategies for biologics. Nat. Rev. Drug Discov..

[74] Sinjari B., D’Ardes D., Santilli M., Rexhepi I., D’Addazio G., Di Carlo P., Chiacchiaretta P., Caputi S., Cipollone F. (2020). SARS-CoV-2 and Oral Manifestation: An Observational, Human Study. J. Clin. Med..

[75] Huang N., Pérez P., Kato T., Mikami Y., Okuda K., Gilmore R.C., Conde C.D., Gasmi B., Stein S., Beach M. (2021). SARS-CoV-2 infection of the oral cavity and saliva. Nat. Med..

[76] Brandtzaeg P. (2010). Function of mucosa-associated lymphoid tissue in antibody formation. Immunol. Investig..

[77] Amorij J.-P., Kersten G.F.A., Saluja V., Tonnis W.F., Hinrichs W.L.J., Slütter B., Bal S.M., Bouwstra J.A., Huckriede A., Jiskoot W. (2012). Towards tailored vaccine delivery: Needs, challenges and perspectives. J. Control. Release.

